# The Benefits of an Interdisciplinary Approach to Mathematics Education on Issues Around Computation in School

**DOI:** 10.3389/fpsyg.2022.533402

**Published:** 2022-06-02

**Authors:** Laura Martignon, Charlotte Rechtsteiner

**Affiliations:** Institute of Mathematics and Computer Science, Ludwigsburg University of Education, Ludwigsburg, Germany

**Keywords:** finger gnosis, number sense, calculation, “Zahlenblick”, flexible mental calculation, operations, natural frequencies, icon arrays

## Abstract

We present arguments in favor of an interdisciplinary approach in mathematics education. As an instance, we briefly recall how cognitive neuropsychologists promoted intense finger gnosis acquisition, i.e., acquiring the ability to mentally represent one’s fingers, at an early age. Mathematics educators definitely recommended the development of finger gnosis but examined its limits. They also presented arguments in favor of developing flexible mental calculation as a goal of arithmetical instruction in elementary school. In this context we describe the training of “Zahlenblick” as a way to foster flexible mental calculation and connect it with concepts from the theory of metacognition. We illustrate how precisely this branch of metacognition demands further interdisciplinary research. In our analysis, “Zahlenblick” extends to acquiring an eye for proportions, beyond just whole numbers. We illustrate how useful it would be to better understand the neural underpinnings responsible for the advantages of so-called natural frequencies, compared with percentages or probabilities, and of icon arrays for representing them. Such natural frequencies are adequate formats for the early confrontation with decision-making under risk.

## Introduction

Dehaene concludes his recent book “How we learn” (2020) with a section on “Reconciling Education with Neuroscience.” This section is an inspirational piece for readers from mathematics education. According to one of the take-home messages in Dehaene’s book, children are all very similar in their learning processes. Dehaene affirms that “The brain circuits for reading and mathematics are the same in each of us, give or take a few millimeters” (Dehaene, 2020, p. 241).

**FIGURE 1:**
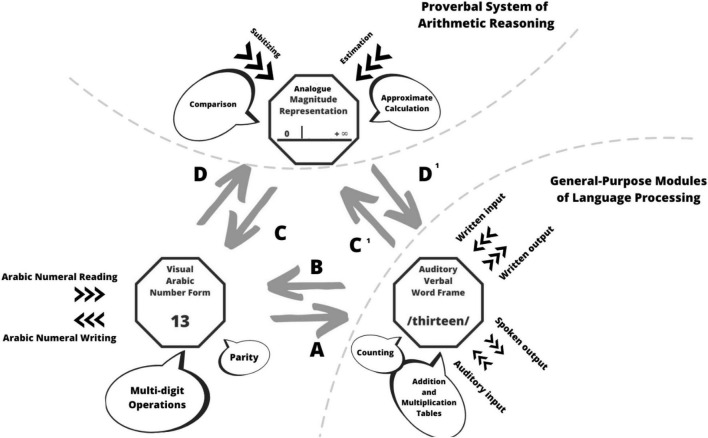
The triple-code model (adapted from Dehaene, 1992, p. 31).

Since the nineties Dehaene’s work has been of fundamental importance for mathematics education. His triple-code model (Figure 1), according to which there are three inter-connected modules in the brain, responsible for our sense for numbers, led to further important discoveries; we will briefly mention a few of them. It also implies important consequences for the treatment of dyscalculia (von Aster, 2000).

Another source of inspiration for mathematics educators in German speaking countries has been the work of Manfred Spitzer. Spitzer bases his theory of learning, among other things, on the so-called hermeneutic circle, according to which prior knowledge is necessary to learn something new in an unstructured environment. Since all actions leave “traces in the brain” (Spitzer, 2008, p. 46)—all the more intensively, the more frequently they are performed—it does matter what children and adolescents do all day. Children learn significantly faster than adults. Acting and grasping (meant in the literal sense) play a role not only in learning concrete individual things, but also in learning general knowledge (semantic memory and even abstract concepts such as numbers). That is why Spitzer advocates finger games instead of laptops in kindergartens and handwritten writing with a pencil instead of typing on a keyboard.

Based on the insights gained from such fertile interactions we report on the dialogue between neuroscientists, cognitive neuropsychologists, and mathematics educators on the use of fingers for representing numbers and the limits of mere finger representations. In particular, we briefly describe progress in the area of ‘‘Zahlenblick’’^1^ training, which aims at guiding children to acquire an eye for numbers, based on a grasp of their structural patterns and properties. “Zahlenblick” refers to the competence to recognize problem characteristics, number patterns and numerical relationships quickly, and to use them for solving problems (Schütte, 2004). In essence, this amounts to an algebraic perspective on numbers and operations with them.

In the context of this algebraic perspective, we will also discuss part-to-whole relationships among numbers, which are the basis for grasping proportions and comparing them. These relationships are fundamental for understanding natural frequencies, which we will discuss in connection with the rudiments of Bayesian Reasoning. These natural frequencies allow for different geometrical visualizations, for instance based on arrays of pictograms or icons called ‘‘icon arrays’’. Icon arrays have been successfully implemented for conveying information on health statistics. Their success has been acknowledged even by insurance companies and risk literacy centers.^2^ Here again gaining comprehension of the neural underpinnings and cognitive processes involved seems fundamental.

## Different or Complementary Perspectives on How Calculation Skills Develop

As we have announced, we propose an interdisciplinary approach to mathematics education. This becomes especially clear when considering the learning of arithmetic. Here, all perspectives have to merge and converge to a fruitful dialogue on children’s learning. At this point we dare to take a first step, which is to be continued in an interdisciplinary discourse.

From the background of the triple-code model (Dehaene, 1992) illustrated in Figure 1, von Aster and Shalev (2007) were able by means of brain imaging procedures to analyze how the mental number line, the verbal and the Arabic number system interact in the development of the number concept. Dehaene’s findings thus have greatly influenced the educational discussion, in which the importance of the empty number line is emphasized as a supplement to the development of cardinal number conceptions as a support for an extensive number concept (e.g., Lorenz, 2005). Inspired by these results a model for a comprehensive number concept development by the second author of this paper (Rechtsteiner-Merz, 2013, p. 58) integrates the different numerical aspects—also the relative location of numbers on an imagined line—as an important component.

In the context of cardinality, finger-based representations of numbers is a precursor ability on which more abstract, symbolic representations of numbers and the development of basic arithmetic can be built throughout life (Wasner et al., 2014, 2016). Evidence for this is provided by findings from cognitive neuropsychological research, which has been influential for strengthening attitudes of mathematics educators toward finger counting in early schooling.

Starting from the association between fingers and numbers, questions about functional relevance have been raised in studies by cognitive neuroscientists (Moeller et al., 2011; Wasner et al., 2016) including the question of how intense the emphasis placed on finger counting and finger arithmetic strategies should be during early schooling. One of the messages has been that “better finger representations imply a lower risk of later poor arithmetic performance” (Moeller and Nuerk, 2012, p. 8). What matters is the relation between this early ability and other early skills that have been shown to be good indicators of later performance in mathematics education. Krajewski (2003, 2005), for instance, was able to detect high correlations between “quantity- and number-related prior knowledge” and mathematical achievement by the end of elementary school: This correlation was discovered in a longitudinal study in which she observed 130 children from 6 months before school entry till the end of the fourth school year. The term “quantity- and number-related prior knowledge” encompasses knowledge about numbers and quantities, as well as counting and initial arithmetic skills. The proven correlation remained unaltered even when it was adjusted for intelligence. In the study 61% of the first graders with poor arithmetic skills could already be identified as “conspicuous” 6 months before school entry on the basis of their knowledge of quantities and numbers; by contrast, only 43% of these children could be predicted by intelligence (Krajewski, 2003).

## Beyond Cardinal and Ordinal Aspects

An important recommendation from cognitive neuroscience has been to propose intervention studies to target finger-based representations of number (size), at least in kindergarten and first grade, to support children’s numerical development (Moeller and Nuerk, 2012). From a mathematics education perspective, the important complementary question has been whether mere finger-based representations can lead to a robust understanding of numerical properties beyond cardinality, i.e., aspects of numbers as ordinals or as codes.

The dialogue promoted a sharpening of research questions on whether number comprehension, operational comprehension, and strategic means,^3^ based on a broad understanding, can be developed on the basis of finger representations and finger counting. Which aspects can be promoted in the development of an understanding of numbers and arithmetic with the help of the fingers, which cannot? While neuroscientific and cognitive-psychological studies often seem to focus exclusively on the correctness of the result (e g., Käser et al., 2013), the declared aim of mathematics education is the development of flexible mental calculation and thus the focus on diverse solution paths from the very beginning (e g., Selter, 2000; Anghileri, 2001; Baroody, 2003; Rathgeb-Schnierer and Green, 2017).

### Developing a Comprehensive Concept of Numbers

From the perspective of mathematics education, cardinal experiences have to result in a comprehensive part-part-whole concept, in the sense of composing/decomposing. This means that children understand how numbers can be decomposed into parts and put together, while the total value remains the same (Gerster and Schultz, 2004). For example, 7 can be expressed as 3 + 4 or 2 + 5, just as 76 can be expressed as 70 + 6 or 60 + 16, and so on. These ideas initially developed in the context of cardinality lead to the basic notion of flexible part-whole relationships, which form the basis for calculating.

**FIGURE 2:**
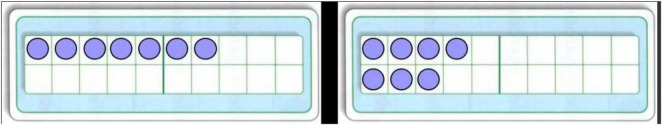
On the left hand side the blocks for decomposing 7 are based on the “strength of *five*,” while the decomposition on the right hand side goes down to a block of 4 dots and the rest, namely 3 dots or 6 dots like the cube and one.

When the numbers up to 10 are represented with the fingers, relationships of closeness to and from 5 and to and from 10 can become clear. However, studies by Gaidoschik (2010) or also by Gerster and Schultz (2004) have shown that even if a child can spontaneously represent a number with his/her fingers, he or she is not necessarily aware of the more general part-part-whole relationships and does not necessarily use it when adding and subtracting. In addition, the row representation of the fingers hardly stimulates any other part-whole relationships besides the focus on the five and the ten, and other representations such as the block representation in the “ten field” must be supplemented for this purpose (Rathgeb-Schnierer and Rechtsteiner, 2018), as illustrated in Figure 2.

Cardinal experiences for grasping, representing, structuring, decomposing, and comparing, which result in a flexible part-whole concept, are central on the way to develop calculation. From a didactical position, supported by the neuroscientific perspective, however, children also need experiences in flexible counting as well as ordinal experiences and experiences of measurement in the sense of the relative positioning of numbers within an interval on an empty number line (e.g., Lorenz, 1997, 2005; Rechtsteiner-Merz, 2013).

### Developing an Insight Into Mathematical Laws and Acquiring Strategic Means for Calculation

Having to mentally add 9 and 8, children can work with decompositions, for instance, as illustrated below:


9+8=9+(1+7)=(9+1)+7=10+7=17

They can also use different auxiliary tasks like 8 + 8, 9 + 9, 10 + 8 which can be viewed as “neighboring tasks,” or they can change the two summands in opposite directions to 10 + 7.

Finding a “convenient” decomposition of numbers or a corresponding auxiliary task for adding two summands is the basis of mental calculation. In the concrete case above, 8 can be “seen” as 1 + 7 and the convenience of this decomposition is also seen as convenient for simplifying the addition with 9. To use these number decompositions and auxiliary tasks requires that children have automated them (see Wessolowski, 2010), in the sense of System 1 in the dual process model (Kahneman, 2011), as we will discuss in section “Connecting the Development of ‘Zahlenblick’ With the Dual ‘Process Model’ for Basic Arithmetic Operations” below. The trick is to reduce more difficult tasks to easier, automatic ones. Many recent studies in mathematics education have been devoted to examining “flexible mental calculation” (e.g., Rathgeb-Schnierer, 2006a; Rechtsteiner-Merz, 2013; Heinze et al., 2015). Can these insights on number de- and compositions be gained from finger representations? The closeness to and anchoring on the numbers 5 and 10 can be seen through finger representations, but the limits are obvious (see above). Also children have to go beyond the range of 10 fingers quite early in school.

According to some cognitive neuroscientists, counting forward and counting backward with the fingers during addition and subtraction illustrates the basic principles of the first arithmetic operations (Moeller and Nuerk, 2012). Here again the vision of mathematics education differs considerably: adding and taking away fingers at the semantic level of arithmetic operations only corresponds to the situation types, in Schipper’s (2009) terminology, of “Changing,” “Combining,” “Comparing,” and “Equating” and are not illustrated by means of finger representations *per se*. Furthermore, the adherence to counting with fingers for too long may even have undesired consequences for learning arithmetic. Concerning the development of arithmetic strategies in the first school year, Gaidoschik (2010, 2019) goes as far as stating that mere counting can even become a conceptual obstacle for going beyond counting.

## Developing Flexible Mental Calculation as a Goal

In mathematics education, the development of flexible mental calculation is considered the central goal of arithmetic mathematics instruction in elementary school (e.g., Selter, 2000; Anghileri, 2001; Verschaffel et al., 2009).

However, regarding flexible mental calculation in detail there are different definitions in current literature (e.g., Verschaffel et al., 2009; Rathgeb-Schnierer and Green, 2013; Rechtsteiner and Rathgeb-Schnierer, 2017). As can be shown, both the research approach and the focus on the fostering of flexible mental calculation depend on the understanding of flexible mental calculation *per se*. All of the above cited research contributions have in common the distinction between two aspects: the shift between different strategic means and the aspect of adaptivity. It is the particular interpretation of “adaptivity” which makes the difference (Verschaffel et al., 2009; Rechtsteiner-Merz, 2013, 2015; Rechtsteiner-Merz and Rathgeb-Schnierer, 2015; Nunes et al., 2016; Rechtsteiner and Rathgeb-Schnierer, 2017). There are two main perspectives:

First: the adaptive use of solution methods is understood as a match between solution methods and problem characteristics. These approaches underlie the assumption that there exists one best or most suitable solution path for each term (see e.g., Klein and Beishuizen, 1998; Blöte et al., 2001; Star and Newton, 2009). As far as rating criteria for this matching are concerned, we find predominantly two different methods in research: (1) accuracy and speed in obtaining a solution (Torbeyns et al., 2005; Verschaffel et al., 2009), and (2) the number of steps (Star and Newton, 2009). When considering adaptivity, this approach focuses on the level “tools for solution” (Rathgeb-Schnierer and Green, 2013, p. 254) (such as the use of basic facts and strategic means) or that of “methods of calculation” (Rathgeb-Schnierer and Green, 2013, p. 254) (such as mental arithmetic, semi-written arithmetic, or the algorithm). For the development of flexible mental calculation this means that the focus is mainly on teaching different strategies and comparing them (e.g., Lorenz, 1997; Klein and Beishuizen, 1998; Verschaffel et al., 2009).

Second: the adaptive use is connected with the recognition of number and problem characteristics during the solution process (Threlfall, 2002, 2009; Rathgeb-Schnierer and Green, 2013; Rechtsteiner-Merz and Rathgeb-Schnierer, 2015; Rechtsteiner and Rathgeb-Schnierer, 2017). Researchers who adopt this approach focus on the match between the combination of strategic means and the recognition of number patterns and numerical relationships—the “cognitive elements” after Rathgeb-Schnierer and Green (2013, p. 254)—of a given problem during the calculation process. This implies for the development of flexible mental calculation that the focus for training must be on both aspects—the cognitive elements and the development of strategic means. The development of “Zahlenblick” is a special approach for mathematics education that places the emphasis on number patterns and numerical relationships, which then results in the development of strategic means.

The proposed steps can be summarized by stating that counting and the use of fingers represent central elements in mathematics education as the first important step of young children toward the understanding of numbers and arithmetic, and that these preliminary experiences are also linked in the sense of an intermediate stage in modern instruction (Schipper, 2009; Hasemann and Gasteiger, 2020; Padberg and Benz, 2021). A number may stand for infinitely many sets and also allow for different decompositions; a finger representation stands for only one of those sets. Children should be able to grasp small numbers spontaneously and larger numbers quasi-spontaneously, which requires “structured representations,” for instance, by means of fingers, decomposing 6 into 5 and 1 or by means of dots of dice by decomposing 6 into 3 and 3 etc. (e.g., Sprenger and Benz, 2020). In addition, it is not only a matter of the quick comprehension of numbers, but in particular—as described above—also the recognition of number relationships, which can only be represented to a limited extent with the help of fingers.

In order to be able to calculate flexibly, a comprehensive understanding of numbers with a pronounced part-whole principle is a prerequisite. In addition, however, the perception of number and task relationships plays a central role—the development of “Zahlenblick”. In turn, the development and use of metacognitive competencies for flexible mental calculation is a necessary element for which interdisciplinary research between neuroscience, developmental psychology and mathematics education would be desirable.

## “Zahlenblick” and Its Development by Means of Suitable Activities

The concept of “Zahlenblick” was introduced by Schütte (2004) and has been accepted and introduced in the literature, maintaining the German term. It means the competence of being able to recognize problem characteristics, number patterns and numerical relationships immediately and automatically, and to use them for solving a problem (Schütte, 2004). It is considered as resulting from development.

### Activities That Stimulate “Zahlenblick”

**FIGURE 3:**
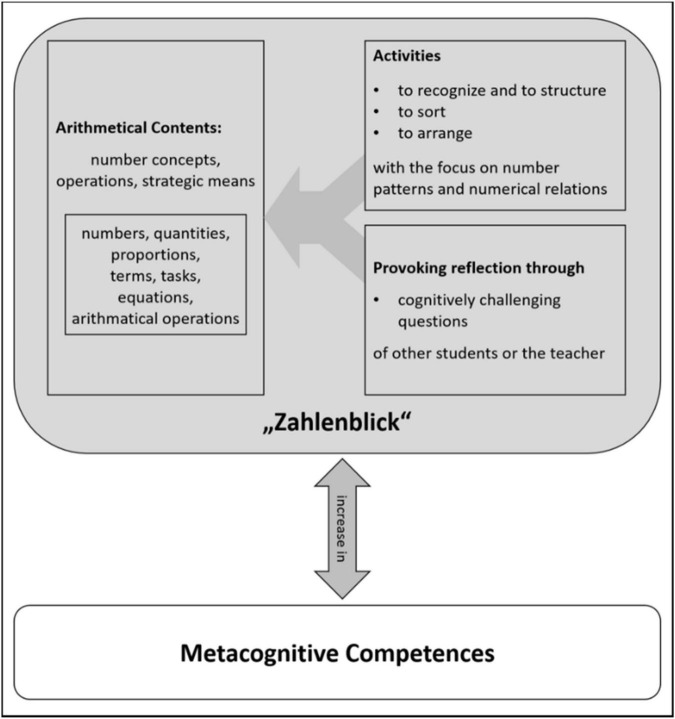
Model for the development of “Zahlenblick” (modified in accordance with Rechtsteiner-Merz, 2013, p. 103).

For the development of “Zahlenblick”, it is crucial to provide activities which highlight the problem characteristics and relationships between problems which target the development of number concepts, understanding of operations and strategic means in elementary school (Figure 3). Therefore, all activities require that students *recognize* and *structure* number patterns, problem characteristics and relations between numbers and problems, and *sort* or *arrange* problems by using structural relations. Additional to this kind of involvement with arithmetical contents, all activities include cognitively challenging questions to provoke students’ thinking and reflection. By combining these two aspects—highlighting number and problem characteristics and provoking students’ reflection—an increase of metacognitive competences concerning the algebraic structure of numbers can be developed (Rechtsteiner-Merz, 2013; Rechtsteiner and Rathgeb-Schnierer, 2017), that is a knowledge of one’s own ability to make use of associativity, commutativity, and distributive laws. At the same time these metacognitive competencies foster the development of “Zahlenblick.”

### “Zahlenblick” and Metacognition

Metacognition has become a buzzword in several realms of mathematics education. Here we want to define the contours of the term before using it. Metacognition, in our framework, can be described as the knowledge of “information-processing skills, as well as knowledge about the nature of cognitive tasks, and of strategies for coping with such tasks” (Schneider and Artelt, 2010, p. 149).

As different researchers have pointed out, metacognition is central to the process of mathematical problem solving (e.g., Verschaffel, 1999; Kramarski et al., 2010) or when using adaptive strategic means (e.g., Schütte, 2004). In the early definition by Flavell (1976) it meant awareness, reflection and control. It had been discovered by Silver (1985) back in the 1980s (1985) that failure or success in mathematical problem solving can be associated with the lack or the presence of metacognitive strategies. It is our position that something quite similar is also the case in flexible mental calculation. For general problem solving and for improving flexible mental calculation, reflecting on the solution process as well as the embedding in algebraic structures is required. Successful instruction requires two factors:

1. Knowledge about one’s own cognition
2. Self-regulation of one’s own cognition

What is intimately connected with metacognition is a conscious categorization of cognitive instances. In the next section we specify necessary steps for this conscious categorization.

### Metacognition and “Zahlenblick” Training—Three Instances

In the following we describe a generic example for every phenomenon that occurs in conjunction with the activities of “Zahlenblick” and show how metacognition can be developed with a algebraic focus.

“The ‘Zahlenblick’ should help to recognise generalisable aspects in situations, to discover structural similarities between already solved and new tasks and to transfer strategic approaches.” (Schütte, 2008, p. 103).

In this context, algebraic thus does not mean working with variables, but rather the perception, use and classification of number and task relationships and the associated consideration of the inherent structures as well as the generalization of the characteristics and transfer to further tasks and contexts (Schütte, 2004; Akinwumni, 2012; Steinweg, 2013; Rechtsteiner and Scheffknecht, in review).

#### Recognizing

**FIGURE 4:**
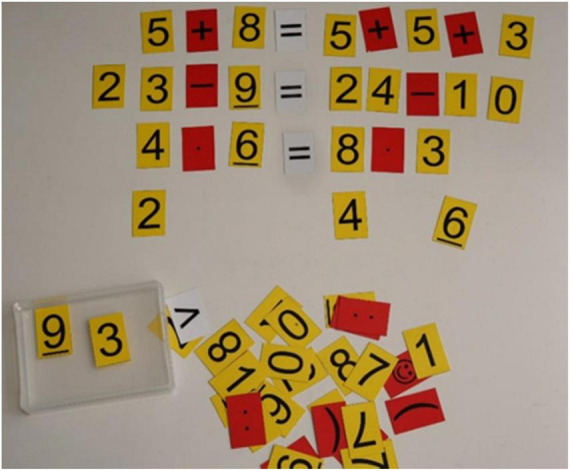
Combining equations by moving cards representing numbers and equalities.

For the activity of “combining equations” (German “Kombi-Gleichungen”), students work with cards with digits on them and other cards with operation symbols, trying to place these cards so as to compose valid equations (Baireuther and Kucharz, 2007; Rechtsteiner-Merz, 2013). This activity is called “combining equations” because digits cards and operation signs of all four basic arithmetic operations are combined in a valid equation resulting from the construction. The aim of the activity is for children to experiment freely and to invent different equations, as in Figure 4.

This activity focuses on promoting algebraic thinking. In contrast to arithmetic, algebra is not about “procedures that lead to a result or a solution” (Steinweg, 2013, p. 3), but “about the relationships between the elements of an equation” (Steinweg, 2013, p. 3). In this way, the “penetration of arithmetic with algebraic ideas (not with formal procedures!)” (Winter, 1987, p. 42) enables a deepening of arithmetic understanding (Rathgeb-Schnierer and Rechtsteiner, 2018).

The subject of equations in primary school teaching aims at developing “concepts” as a connection between arithmetic (procedures) and algebraic ways of thinking (concepts) (Gray and Tall, 1994; Rechtsteiner-Merz, 2013; Steinweg, 2013). The majority of children interpret the equal sign exclusively as a request to calculate and thus operationally as an assignment sign. By dealing with equations, children can also become aware of the relational side of the sign “=” as the equality between the two terms (Steinweg, 2013). Accordingly, in algebraic thinking, terms are not primarily understood as a calculation request, but rather as objects (in terms of their value) to be compared. The focus is on the “algebraic equilibrium view” (Winter, 1987, p. 42) as the equivalence of two terms.

Small changes in the equations raise questions such as “What happens if.?” Or “How can this be balanced?” “How do I get the equation back into balance?” “Why is that?” etc. This opens up first approaches to functional relationships in equations. The goal is to make these comparisons without calculating the respective results. Rather, relationships between the two terms and strategic tools such as mutually opposing changes or disassembling should be used in the comparison. As described above, this strives for an algebraic view that enables students to recognize both algebraic concepts and relationships at a higher level and thus achieve mathematical awareness at the metacognitive level.

#### Sorting

This activity emphasizes sorting tasks—addition and subtraction as well as multiplication and division—into subjective or objective categories (Rathgeb-Schnierer, 2006b) like for example “I need to count it,” “I know it” and “I know a trick to solve it” or “in a group of ten,” “exactly ten” or “amongst a group of ten.”

When introducing the activity, students are encouraged to sort each individual problem into a suitable category without actually solving it yet. Their focus, therefore, is concentrated on problem characteristics as well as numerical relations, and how to categorize them. While discussing with other students and the teacher and comparing their sorting results, they might discover new patterns, new relations or new strategic means. Supplementary, they could think up more problems which can be solved with the same strategic means. For example, they find out that 36 + 29 can be solved by considering 36 + 30—1, which they should find easy. One ideal activity is then for them to generate tasks themselves and propose solutions.

This activity aims at getting students to think about problem characteristics on a more general—algebraic—level, for instance: “What is the reason for assigning several different problems to one category?”; “Do all the problems in one category have similarities?”; “Are there some problems in the category ‘I need to count it’ which are similar to those in the category ‘I already know a trick’?”; “Is it possible to use problems already known by heart to facilitate the solution of problems from the other categories?”; “Why can these problems be solved in the same way?” or “What is the common problem characteristic?” The focus on these algebraic relationships leads to an increase in specific metacognitive competences that enables students to develop clusters of problems, to think about strategic means and their particular specifics, to realize their own competences when operating with them.

#### Arranging

**FIGURE 5:**
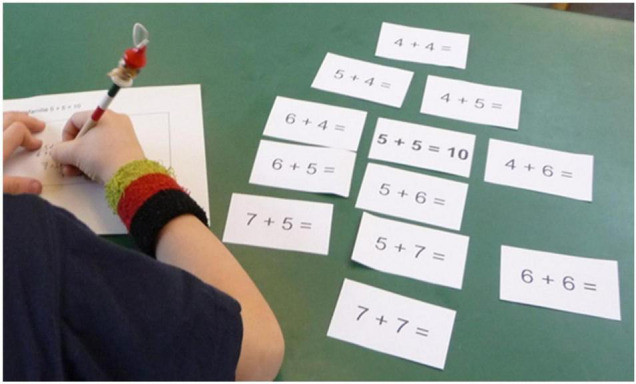
Arranging a “Problem-Family” by placing terms together depending on their relationship.

A “problem-family” is described as a set of structurally related problems (Figure 5). This activity intends to place the emphasis on structural relations between a set of problems (Rechtsteiner and Rathgeb-Schnierer, 2017). First, the students receive one problem with its solution (e.g., 5 + 5 = 10). In a second step, they are asked to arrange multiple cards with related problems (e.g., 5 + 6, 6 + 6, 4 + 6 etc.) around this first one with the aim of making the relationships visible and to explain them. Subsequently, the students are encouraged to discuss their arrangements with others, and give reasons for their decisions. This activity does not focus on solving problems but on the recognition of problem features and relationships. Within this activity, students discover relationships between problems (Rechtsteiner and Rathgeb-Schnierer, 2017). As in the other activities described above, the focus here is also on the structure of these problems for understanding mathematical semantics in an algebraic way. By discussing these coherences students become aware of their own approach and those of the others that allows them to compare and to develop what can be described as metacognition.

### Connecting the Development of “Zahlenblick” With the Dual “Process Model” for Basic Arithmetic Operations

In his book “Thinking Fast and Slow”, Kahneman (2011) revealingly uses a mathematical task, namely multiplying two-digit numbers for illustrating what he and several other cognitive psychologists see as mental actions performed by the so called “System 2.” This is a slow and deliberative system of the mind-brain, which is seen as a contrast to “System 1,” based on quick and intuitive procedures. Multiplying 19 by 24, for instance, be it mentally or operating with paper and pencil, requires activating modules which have been developed in school, even if the brain substrates for such operations pre-exist. A typical implementation of “Zahlenblick” would be to see that 19 = 20-1, and then multiply 20 and 24, and subtract 19 from the result. Mathematics education tends to agree that children should learn at least multiplication tables “by heart” once they have understood basic connections (e.g., Sherin and Fuson, 2005; Gaidoschik, 2014), to the point, as we may add, that 20 times 24 can be rapidly performed by System 1, in Kahneman’s sense.

The emergent view on the relevance of finger representation in the early years in kindergarten may also be expressed by means of Kahneman’s dual system dichotomy: finger gnosis fosters an automatic handling of numbers with System 1, whereas the other components of a good arithmetic instruction in elementary school are designed to foster the development of more meta-cognitive strategies and “Zahlenblick” performed by System 2, which coordinate and use results produced by System 1.

Palm (2016) seems to have been the first to have introduced a compelling metaphor for viewing System 1 and System 2 as an orchestra and its conductor. To state that we have a brain for numbers, to use Andreas Nieder’s title of his inspiring book (2019), means that the musicians in the orchestra can, in fact, play their instruments. But that is not enough for performing music well. The orchestra without the conductor ends up sounding dysfunctional when interpreting a piece of music.

## From Grasping Whole Numbers to Grasping Proportions

While elementary arithmetic is necessary for shopping and paying bills of all sorts, a minimum understanding of proportions becomes handy when we make simple inferences. Individuals need to combine the arithmetic of natural and whole numbers with the arithmetic of proportions for dealing with risks and making good decisions under uncertainty. In mathematics education the discoveries of cognitive psychology have led to the conclusion that, while probabilities are difficult for most people to grasp, elementary proportions, in the guise of so-called “natural frequencies” (Hoffrage et al., 2015) can provide a “natural,” heuristic approach to dealing with risks and uncertainties.

### Fostering Inferences Under Uncertainty: Which Formats Are Useful?

The inception of basic elements of probabilistic inference in primary school is becoming a common feature of school curricula throughout the world, although there are important differences in the time slots allotted to the topic. The first author of this paper, influenced by the work of the ABC Group on the advantages of “ecologically rational” representations, has investigated the effect of simple proportions like “3 out of 4” instead of 0.75 or 3/4 in the classroom when dealing with probabilistic tasks (Martignon and Hoffrage, 2019). Atmaca and Martignon conjectured that different neural circuits are involved when someone solves a probabilistic task based on proportions, or so-called natural frequencies, and when the task is expressed in probabilities. They used a “result verification” or “result disparity” (Kiefer and Dehaene, 1997) experimental frame: subjects are presented with a proposed solution and asked to judge, as quickly as possible, whether it is correct or incorrect. Atmaca and Martignon (2004) found that subjects needed significantly longer times and produced significantly fewer correct answers, for the tasks given in probability format versus those given in the natural frequency format (Atmaca and Martignon, 2004; Martignon et al., 2007). The recent work by cognitive neuroscientists on proportion comparisons is relevant for our discussion: there are neural correlates for the processing of proportions which differ from the typical neural correlates associated with processing of decimals (Mock et al., 2018; Reinhold et al., 2020). More precisely, part-whole relations presented in dot patterns and pie-charts appear to involve neural processes which differ from those involved in the processing of decimals (Jacob and Nieder, 2009). Probabilities are usually expressed in terms of decimals, but their meaning goes far beyond their decimal representations. Our interest here lies in the advantages of icon arrays for representing natural frequencies.

### Icon Arrays: Sorting and Arranging

Icon arrays are graphical representations inspired by Neurath’s (1933/1973) and Isotypes (1933). An icon array is a form of pictograph which uses grids of squares, faces, or other symbols to represent statistical information.

The next example of the use of icon arrays for describing a medical situation exhibits their function. It also illustrates how “sorting and arranging” can be useful in the context of natural frequencies. Consider a fictitious population of 100 people, who have been tested as to whether they suffer from a disease (in this case, the disease considered is HIV, but it could be COVID-19 pandemic, for instance, which was so present while this paper was written). The possible outcomes are:

• no disease and test is negative
• no disease and test is positive
• presence of the disease but test is negative
• disease is present and test is positive

**FIGURE 6:**
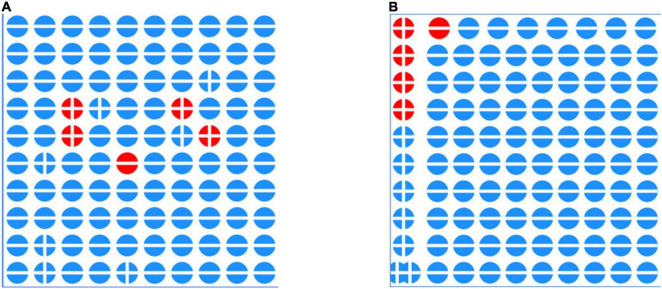
**(A)** The icon array displays icons for 100 people tested as to whether they are HIV positive; unstructured data. **(B)** The same icon array has now been sorted grouping icons representing patients with/without the disease. (These illustrations are all from the dynamic webpage by the first author of this paper and T. Erickson: http://www.eeps.com/projects/wwg/wwg-en.html).

The icons used for representing patients are dots: red dots and blue dots stand for patients with and without the disease, while the “plus” sign denotes a positive test, and the “minus” sign denotes a negative test. The icon array in Figure 6A represent cases as they are treated by a doctor, without any particular ordering, while the icon array in Figure 6B has been sorted so that positive tests are easy to be counted.

**FIGURE 7:**
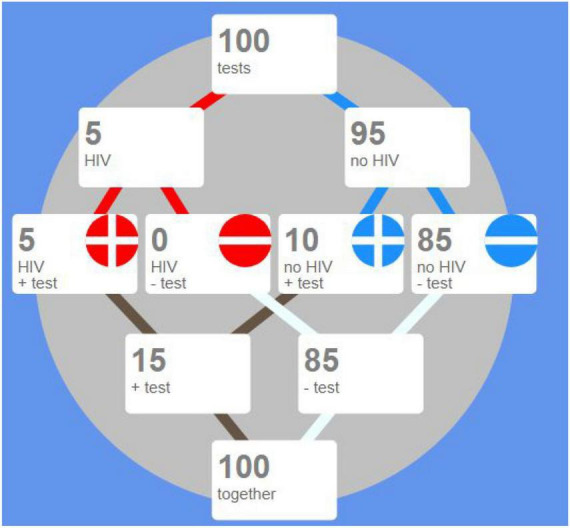
A double tree for inference on the HIV data.

Arranging data for inferential reasoning may be carried out by means of so-called double trees for inference:

The upper half of the display in Figure 7 represents a causal tree, i.e., from having/not having the disease to having positive/negative test results. The lower half of the display represents a diagnostic tree, i.e., from test results to presence or absence of the disease.

These representations are part of a dynamic webpage which the first author has created with Tim Erickson^4^ and which can be reached by activating the QR Code:


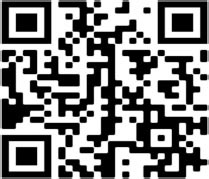


## Conclusion

The examples of fruitful cooperation between researchers from three different disciplines, namely psychology, neuroscience and mathematics education, which we described in the course of this paper, have motivated our search for even stronger interactions. We are not alone in our endeavor. The work of colleagues in neuroscience and cognitive psychology along the same lines also seems encouraging.

The results show, for instance, that the educational goal of developing flexible computational competencies with numbers and proportions—both promoted by the development of Zahlenblick -could be a joint project: Neuroscientific and cognitive psychological perspectives in cooperation with math-educational ones could foster the development of metacognition in connection with the training of Zahlenblick. We are convinced that only an interdisciplinary approach to the basic questions on how the human mind learns to master mathematics will ever provide satisfactory answers to how mathematics is perceived by school students and thus improve the educational skills of future teachers.

## Author Contributions

Both authors listed have made a substantial, direct, and intellectual contribution to the work, and approved it for publication.

## Conflict of Interest

The authors declare that the research was conducted in the absence of any commercial or financial relationships that could be construed as a potential conflict of interest.

## Publisher’s Note

All claims expressed in this article are solely those of the authors and do not necessarily represent those of their affiliated organizations, or those of the publisher, the editors and the reviewers. Any product that may be evaluated in this article, or claim that may be made by its manufacturer, is not guaranteed or endorsed by the publisher.

## Footnotes

1 The concept of “Zahlenblick” was introduced by Schütte (2004) and has been accepted and introduced in the literature, maintaining the German term, which appears to be impossible to translate in just one word.

2 See, for instance, brochures and other materials by the Harding Center https://www.hardingcenter.de/en.

3 Strategic means are distinct devices for modifying problems to make them easier to solve. They can be flexibly combined in a solution process and include, for instance, composing and decomposing, modifying a problem, deriving the solution from a known fact, and using analogies (e.g., Rathgeb-Schnierer, 2006a,^2010^; Rathgeb-Schnierer and Green, 2013).

4
http://www.eeps.com/projects/wwg/wwg-en.html
